# Differential gene expression in nearly isogenic lines with QTL for partial resistance to *Puccinia hordei *in barley

**DOI:** 10.1186/1471-2164-11-629

**Published:** 2010-11-11

**Authors:** Xinwei Chen, Rients E Niks, Peter E Hedley, Jenny Morris, Arnis Druka, Thierry C Marcel, Anton Vels, Robbie Waugh

**Affiliations:** 1 Genetics Programme, Scottish Crop Research Institute, Dundee, UK; 2 Laboratory of Plant Breeding, Graduate School for Experimental Plant Sciences, Wageningen University, Wageningen, The Netherlands; 3 INRA-AgroParisTech, UMR 1290 BIOGER-CPP, Avenue Lucien Brétignières BP01, 78850 Thiverval-Grignon, France

## Abstract

**Background:**

The barley-*Puccinia hordei *(barley leaf rust) pathosystem is a model for investigating partial disease resistance in crop plants and genetic mapping of phenotypic resistance has identified several quantitative trait loci (QTL) for partial resistance. Reciprocal QTL-specific near-isogenic lines (QTL-NILs) have been developed that combine two QTL, *Rphq*2 and *Rphq*3, the largest effects detected in a recombinant-inbred-line (RIL) population derived from a cross between the super-susceptible line L94 and partially-resistant line Vada. The molecular mechanism underpinning partial resistance in these QTL-NILs is unknown.

**Results:**

An Agilent custom microarray consisting of 15,000 probes derived from barley consensus EST sequences was used to investigate genome-wide and QTL-specific differential expression of genes 18 hours post-inoculation (hpi) with *Puccinia hordei*. A total of 1,410 genes were identified as being significantly differentially expressed across the genome, of which 55 were accounted for by the genetic differences defined by QTL-NILs at *Rphq*2 and *Rphq*3. These genes were predominantly located at the QTL regions and are, therefore, positional candidates. One gene, encoding the transcriptional repressor Ethylene-Responsive Element Binding Factor 4 (*HvERF4*) was located outside the QTL at 71 cM on chromosome 1H, within a previously detected eQTL hotspot for defence response. The results indicate that *Rphq*2 or *Rphq*3 contains a *trans*-eQTL that modulates expression of *HvERF4*. We speculate that HvERF4 functions as an intermediate that conveys the response signal from a gene(s) contained within *Rphq*2 or *Rphq*3 to a host of down-stream defense responsive genes. Our results also reveal that barley lines with extreme or intermediate partial resistance phenotypes exhibit a profound similarity in their spectrum of *Ph*-responsive genes and that hormone-related signalling pathways are actively involved in response to *Puccinia hordei*.

**Conclusions:**

Differential gene expression between QTL-NILs identifies genes predominantly located within the target region(s) providing both transcriptional and positional candidate genes for the QTL. Genetically mapping the differentially expressed genes relative to the QTL has the potential to discover *trans*-eQTL mediated regulatory relays initiated from genes within the QTL regions.

## Background

Plants have evolved complex mechanisms to defend against pathogen attack. Two types of immunity have been described: Pathogen-Associated Molecular Pattern (PAMP)-Triggered Immunity (PTI) and Effector-Triggered Immunity (ETI). PTI is induced at an early stage when PAMPs are recognized by Pattern Recognition Receptors (PRRs), whereas ETI is induced by direct or indirect association of a Resistance (R) protein with a pathogen-derived effector [1-4]. The outcomes of the two immune systems appear to be partial or quantitative resistance and non-host resistance (PTI), and qualitative resistance (ETI). Recently, Niks and Marcel [5] proposed that the varying efficacy of PTI suppression by pathogen effectors may explain partial resistance. In cereal crops, the barley-*Puccinia hordei *Otth (barley leaf rust) pathosystem is a model for investigating partial and non-host resistance. Microscopic studies on resistance levels in relation to the pathogen developmental phases has indicated plant cell wall penetration and haustorium formation by *P. hordei *as critical phases determining the success or failure of the infection [6]. Pre-haustorial resistance reduces the chance of successful haustorium formation by the fungal pathogen in the host cells. Failed attempts are typically associated with cell wall appositions [6-10]. Such pre-haustorial basal host defence is a typical reaction to *Ph*-infection in most (if not all) barley lines exhibiting partial resistance [6]. Post-haustorial resistance is usually due to *R *gene-mediated hypersensitive response after haustorium formation [9].

These two types of resistance have strategic significance in plant breeding for resistance to diseases. Quantitative or partial resistance has become increasingly important because of its broader spectrum and higher durability compared to *R*-gene mediated race-specific resistance. Many of the genes underlying partial resistance have plant developmental stage-dependent effectiveness [11]. Currently, over 20 quantitative trait loci (QTL) for quantitative basal resistance to leaf rust from five different mapping populations have been mapped to barley chromosomes [11-16]. They are named *Rphq *genes [Resistance to *Puccinia hordei *(quantitative)]. Of these, 10 were effective during the seedling stage, and were detected by QTL analysis of the latency period exhibited by the rust fungus on seedling leaves [15]. Considerable effort has been expended in an attempt to identify the genes underlying these QTL. Notably, a set of NILs and reciprocal NILs have been developed that contain single (*Rphq*2, 3, 4) or combined (*Rphq*2*+*3) introgressed segment(s) carrying resistance and susceptibility QTL allele(s) that were identified in an L94 × Vada RIL population [11,16,17]. L94 is an Ethiopian landrace and highly susceptible to barley leaf rust. Vada is a Dutch cultivar expressing a high level of partial resistance. Following a positional cloning strategy, Marcel *et al*. [18] have fine-mapped *Rphq*2, the QTL with largest effect, to an interval of 0.11 cM corresponding to less than 200 kb in physical length.

Microarray technology is being widely used to address various biological, biochemical and genetic questions. Microarray-based gene expression studies can be generally grouped into two major categories. The first aims to address specific biological questions by monitoring the differential expression of genes under contrasting conditions or over time. The most common studies in this field are the investigations on host-pathogen interactions. Profiling changes in genome-wide expression in response to pathogen challenge has identified a large spectrum of genes that are responsive to pathogen attack or are associated with plant resistance in various pathosystems (reviewed by Wise *et al*. [19]). The second category is based on the more recently emerged concept of 'genetical genomics' [20] or expression QTL (eQTL) mapping that combines highly parallel gene expression studies with the power of genetic segregation. eQTL studies have been performed on maize, eucalyptus and *Arabidopsis *[21]. eQTL analyses in barley have addressed the global genetic architecture of transcript abundance in 

[22], the phenomenon of limited pleiotropy [23] and as an approach to identify the causal or candidate genes underlying partial resistance to fungal diseases [24,25]. While both categories of microarray studies are based on variation in transcript abundance, eQTL analysis provides a genetic dimension that can differentiate *cis-*from *trans-*regulation and the genetic locations of a large number of genes through the co-location of high LOD eQTL (*i.e*. highly differentially expressed) and their structural genes [26]. This is particularly valuable for a crop with large and unsequenced genome like barley.

Here, using a previously reported Agilent 15 k custom array [25], we performed differential expression analysis of QTL-NILs and their recurrent parental lines at 18 hours post-inoculation (hpi) with *Puccinia hordei*. Our major objective was to identify candidate genes for *Rphq*2 and *Rphq*3. In addition, transcript profiles between *Ph*-infected parents and their respective mock-inoculated controls allowed the establishment of transcriptomic signatures for each line in response to *Ph*-infection. Our results indicate that transcriptional differentiation between QTL-NILs and their respective recurrent parents reveals components of a regulatory transcriptional relay induced in response to *Ph*-infection. The datasets generated offer a basis for further studies on defence signalling in relation to partial resistance to *P. hordei *in barley.

## Results

### Transcriptomic signatures of response to *P. hordei *(Ph infected vs. Mock inoculated)

**Figure 1:**
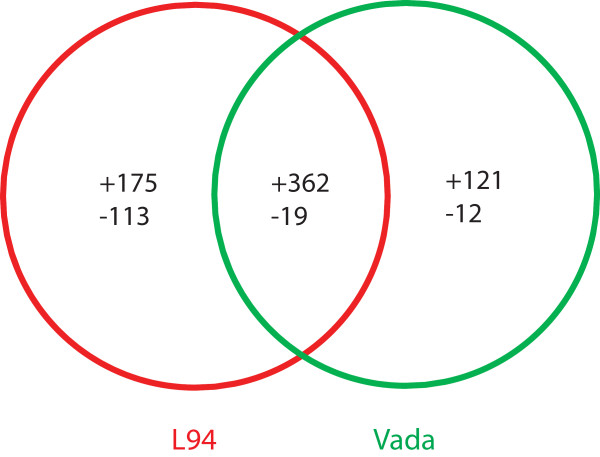
**Venn diagram showing number of *Ph*-responsive genes (fold change >2, FDR <0.05) identified in L94 and Vada**. '+' and '-' represent up- and down-regulation respectively.

**Figure 2:**
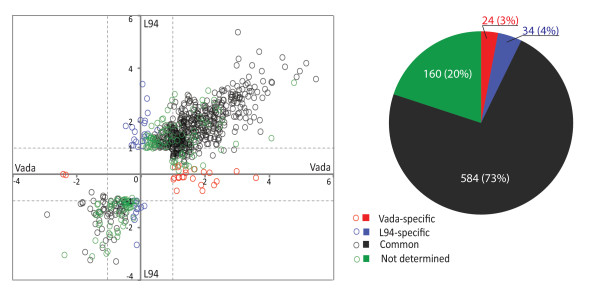
**Scatter plot of log ratios (ratio of signal intensity *Ph*-infected/Mock control) of the 802 *Ph*-responsive genes from Vada (horizontal axis) and L94 (vertical axis)**. Colour-coded circles represent genes in different groups with proportions shown in the pie chart. Log ratios >0 or <0 indicates up- or down-regulation respectively, dashed lines set at 1 and -1 corresponding to 2× fold change in expression.

**Figure 3:**
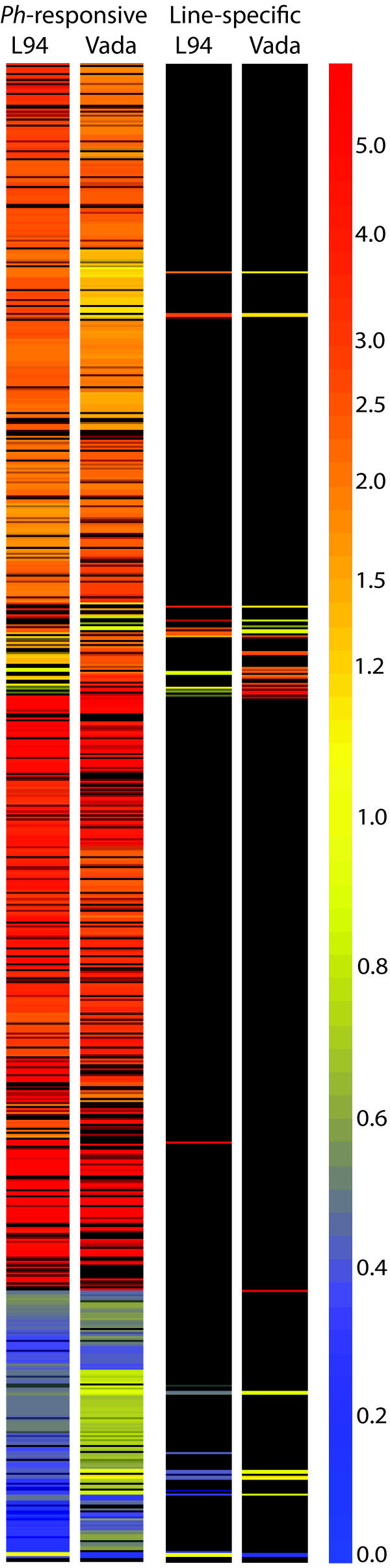
**A heat map illustrating expression patterns of the 802 *Ph*-responsive genes identified in L94 and Vada**. Genes are organized by 'gene tree' hierarchical clustering implemented in GeneSpring based on overall similarity in expression patters (the gene tree has been omitted for clarity). The color bar indicates the expression ratios of the two treatments (*Ph*-infection *vs*. mock-inoculated controls). Red and blue represent up- and down-regulation respectively, whereas yellow represent no significant alteration. Left panel shows 802 genes that were significantly (FC >2, FDR <0.05) altered in at least one of the two lines; right panel shows the 58 line-specific genes that were only significantly (FC >2, FDR <0.05) altered in one line but not the other.

Plant defence responses involve transcriptional activation of a plethora of specific genes and regulation of their temporal and spatial expression [27]. To investigate the genome-wide transcriptional signatures of susceptible and partially resistant barley lines L94 and Vada respectively in response to *P. hordei *infection, we compared *Ph*-infected with mock-inoculated leaf material. A stringent threshold with fold change >2 and false discovery rate (FDR) <0.05 was adopted for declaring significant differences. At this threshold, 669 and 514 genes were respectively identified in L94 and Vada as 'significantly differentially expressed' with 381 (362 up + 19 down) overlapping between the two lines while 421 (L94 175 up +113 down and Vada 121 up + 12 down) were present in only one of the two parents (Figure 1). This yielded a total of 802 genes which we considered '*Ph*-responsive'. Close examination of the expression data of the 421 '*Ph*-responsive' genes from both parents showed that while a substantial number failed to meet the stringent thresholds applied (fold change >2, FDR <0.05) they still exhibited statistically significant differential expression in both parents. Therefore, a relaxed threshold ignoring the fold changes was adopted for the follow-up analysis on the commonality and specificity of response to *Ph*-infection between the resistant and susceptible lines using all 802 *Ph*-responsive genes. We plotted the log-transformed expression ratios of Vada against L94 and classified them into four groups. Genes that showed the same expression patterns (up- or down-regulation) and expression changes at *p *< 0.05 in both lines were defined as being common to both lines (Figure 2, black empty circles), whereas, those that showed significant expression changes in one line but no significant expression changes (*p *> 0.5) or a contrasting expression pattern in the other line were considered as being line-specific (Figure 2, red empty circles for Vada and blue for L94). The remaining genes that had no strong evidence to suggest either commonality or specificity were grouped into 'not determined' (Figure 2, green empty circles). There were a total of 584, 24, 34 and 160 genes that appeared to be in common, Vada- or L94-specific or 'not determined' representing 73%, 3%, 4% and 20% of the 802 *Ph*-responsive genes respectively. Figure 3 shows a colour-coded heat map that was converted from the relative expression ratios (signal intensity from *Ph*-infected *vs*. mock-inoculated controls) of the 802 genes and the 58 (24 + 34) line-specific genes showing the overall similarity and specificity of gene expression in L94 and Vada. Full expression information of the 802 genes and the line-specific genes is given in the Additional File 1 (Table S1) and 2 (Table S2) respectively.

Genome-wide *Ph*-responsive genes have previously been investigated [25] using Steptoe (St) and Morex (Mx), two barley cultivars with similar, intermediate levels of resistance to *P. hordei *(leaf materials were prepared from the same experiment as the current study with L94 and Vada). We therefore compared the data from L94/Vada with those from St/Mx. At exactly the same thresholds (*i.e*. FC >2, FDR <0.05) a total of 1154 genes were identified as *Ph*-responsive in St/Mx [25]. Applying exactly the same criteria as described above, we identified 913 (79%), 21 (1.8%), and 19 (1.6%) genes that were common, St-specific and Mx-specific respectively. We then explored the common genes in each of these categories between the two experiments (Table 1). 75.4% (605) of the 802 genes detected with L94/Vada were also detected with St/Mx and more than half of the genes (466) were significant in all four lines, highlighting the similarity of response to *Ph*-infection across genotypes. Of the 24 and 34 genes that were specifically detected in Vada and L94 respectively, 13 and six of these were reproducibly identified as *Ph*-responsive in Steptoe or Morex (Additional File 3, Table S3). All of the 13 Vada (resistant)-specific genes were up-regulated in Vada and St or Mx. Ten of these genes showed significant differential expression (*p *< 0.05) between St and Mx. Of the six L94 (susceptible)-specific genes, only one up-regulated gene (unigene21775) showed significant differential expression between St and Mx (Additional File 3, Table S3).

**Table 1:** Number of overlapping genes (shown in matrix) in different categories detected in two experiments with St/Mx and Vada/L94

	Ph-responsive (1154)	Common (913)	St-specific (21)	Mx-specific (19)
Ph-responsive (802)	605	532	9	5
Common (584)	506	466	5	3
Vada-specific (24)	13	6	3	2
L94-specific (34)	6	3	0	1

Note: *Ph*-responsive genes were selected on criteria with fold change >2 and FDR <0.05; genes with similar patterns were selected from *Ph*-responsive gene on *p *< 0.05 without considering fold changes.

**Figure 4:**
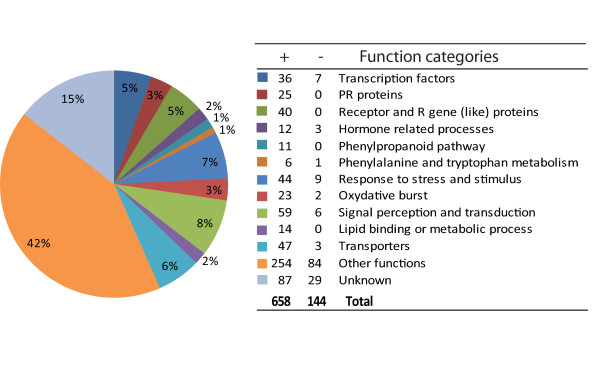
**Functional classification of the 802 *Ph*-responsive genes**. Number of up (+) or down (-) regulated genes are shown in the table (see Additional File 1, Table S1 for details).

To further characterise the biological processes represented by the 802 *Ph*-responsive genes, we performed gene ontology (GO) analysis by classifying the *Ph*-responsive genes into functional biological categories based on GO terms retrieved from their rice homologues through the rice database at http://rice.plantbiology.msu.edu/annotation_pseudo_goslim.shtml. The *Ph*-responsive genes were associated with a broad range of biological processes. The primary category was related to defence response. We further classified these genes into 11 major functional categories following the GO terms in 'biological process' with all remaining genes grouped into 'other functions' or 'unknown'. The results are shown in Figure 4. They indicate that at the sampling time point of 18 hpi, the plants had responded to defend against the *Ph*-infection.

### Differential expression analysis between Ph-infected recurrent parents (L94 vs. Vada)

We performed genome-wide differential expression analysis by comparing expression differences between *Ph*-infected L94 and *Ph*-infected Vada at 18 hpi. A total of 1411 genes were identified as being differentially expressed (FC >2, FDR <0.05), of which 247 were *Ph*-responsive genes as described above. The majority (1164) represent genome-wide, genotype-specific differences in gene expression. The detailed information of these genes regarding their expression ratios, *p*-values and functional annotations is presented in Additional File 4 (Table S4).

### Differential expression between *Ph*-infected QTL-NILs and *Ph*-infected recurrent parents

**Figure 5:**
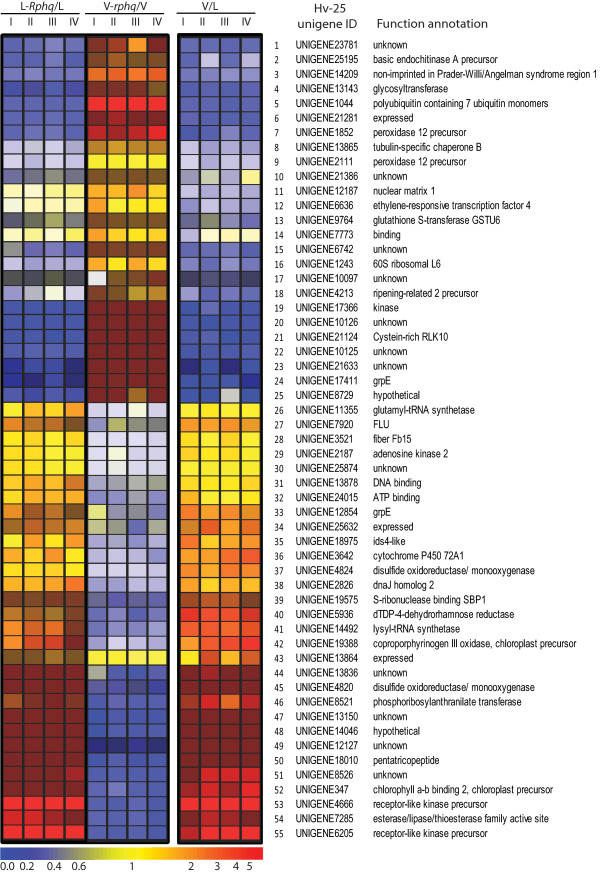
**Heat map of the genes significantly and differentially expressed in the three comparisons**. 'L' and 'V' on top of the heat map refer to L94 and Vada respectively. Roman numerals represent the four biological replicates. Colour coding represents the transcript abundance ratios. The two comparisons involving NILs were performed on microarray slide 3 and showed reversed colouring reflecting the reciprocal features of the NILs in their genetic background. Comparison between the two parents was conducted on microarray slide 2 with transcript abundance being calculated as L94/Vada. The genes (rows) and treatment groups (columns) are clustered through gene tree generation by GeneSpring program on distance (gene tree has been omitted for clarity).

To identify QTL-specific and differentially expressed genes accounted for by genetic differences in the QTL regions, the two reciprocal QTL-NILs were compared with their respective recurrent parents: L94 *vs*. L94-*Rphq*2*+*3 and Vada *vs*. Vada-*Rphq*2*+*3. A total of 94 genes were identified as significant (FC >2, FDR <0.05) in at least one comparison. Of these, 39 genes showed a significant difference in one recurrent parent/QTL-NIL comparison but not with the other. We attribute these observations to the different size and incomplete overlap of the introgressed segments in the two recurrent parent/QTL-NIL pairs. These genes were, therefore, not pursued further. The remaining 55 genes showed expression differences at *p *< 0.05 in both comparisons and were, therefore, considered potentially relevant to the QTL regions. This suggests that differential expression results from genetic factors differing specifically within the QTL regions (Additional File 5, Table S5, and Figure 5). Of these 55 genes, 50 were present on the list of 1411 differentially expressed genes between *Ph*-infected Vada and *Ph*-infected L94. The remaining five genes (Table S5, underlined) did not fulfil the criteria (FC >2, FDR <0.05) set for the differential expression between the two *Ph*-infected recurrent parents, but their expression differences were still statistically significant (*p *< 0.05).

### Transcription of QTL-specific and differentially expressed genes in response to *Ph*-infection

To identify whether the 55 QTL-specific and differentially expressed genes were also *Ph*-responsive, expression data from the *Ph*-infected *vs*. Mock-inoculated experiment was re-investigated (Table S5). Six genes showed changes that fulfilled the criteria (fold change >2, FDR <0.05) set for defining *Ph*-responsive genes. Twelve genes did not fully meet the criteria, but their level of differential expression was still statistically significant (*p *< 0.05) in at least one of the lines. The others were not statistically significant.

### Identification of positional candidates for *Rphq2 *and *Rphq3*

**Figure 6:**
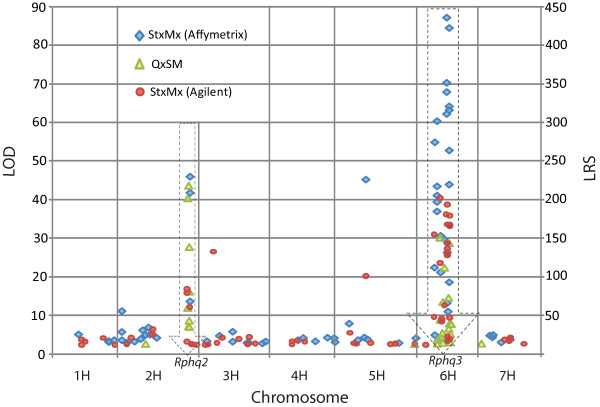
**Distribution of the 163 eQTL detected from three experiments for the 52 genes (3 genes without eQTL detected) differentially expressed in QTL-specific NILs**. Blue diamond, red dots and green triangles represent eQTL identified by Potokina *et al*. [22], Chen *et al*. [25] and Wise *et al*. (unpublished results) respectively. eQTL co-located with *Rphq*2 and *Rphq*3 were framed with dash-lined arrows. Significance levels of eQTL detected in the St/Mx population refer to LOD score, those with 'Q × SM' population refer to LRS.

To determine the map position of the 55 QTL-specific and differentially expressed genes, we took advantage of available datasets previously generated in three different eQTL studies (germinating embryos [22]; *P. tritici *infected leaves http://genenetwork.org, R. Wise, unpublished data) and *Ph*-infected seedling leaves [25]. 52 of the 55 genes had one or more eQTL in at least one of these three experiments, yielding 163 eQTL in total. The distribution of these eQTL was investigated by plotting their map positions against their LOD/LRS values (Figure 6). 40 genes with eQTL mapped to within the QTL regions (nine at *Rphq*2 and 31 at *Rphq*3) (Figure 6, Table S5), of which 33 (83%) had LOD >10 or LRS >50 suggesting they are *cis-*eQTL (*i.e*. their structural genes map to the same locus as the eQTL). We then explored three available gene-based mapping datasets: Illumina OPA-SNPs [28], Single Feature Polymorphisms (R. Wise, unpublished data) and TDMs [22] to help assign genetic map positions to the 55 genes. This allowed four and nine genes to be placed within the confidence intervals of *Rphq*2 and *Rphq*3 respectively. All of these genes overlapped with the eQTL except two (unigene7920 and 2826) for which no eQTL was detected in the three eQTL studies (Table S5). *Rphq*2 and *Rphq*3 on chromosome 2H and 6H are syntenic to regions on rice chromosomes Os04 and Os02 respectively. Conservation of synteny allowed us to infer the approximate map positions of an additional 15 genes to within the QTL regions (Table S5). Thus, of the 55 QTL-specific differentially expressed genes, the map location of nine and 34 fell within *Rphq*2 and *Rphq*3 respectively, whilst the 11 others remain unknown. Of note was the observation that one gene (unigene6636), encoding an Ethylene-Responsive Transcription Factor 4 (HvERF4) (rice orthologue Os05g41780.1), has been mapped as Illumina OPA-SNP marker 11_10686 to chromosome 1H at position 71 cM [28]. This map position is consistent with a location based on conservation of synteny between rice Os05 and barley chromosome 1H, suggesting that differential expression of this gene is the consequence of *trans*-regulation by a gene located within either *Rphq*2 or *Rphq*3.

## Discussion

In this study, we performed differential expression analysis of two reciprocal QTL-NILs and compared them with their respective recurrent parents. As QTL-NILs differ genetically from their recurrent parent only in the selected QTL regions, we would anticipate that genetic polymorphism between these QTL regions would account for any differential expression observed. However, due to the complexity of gene regulation, differentially expressed genes may not necessarily be located in the introgressed QTL regions, which may themselves contain regulatory genes affecting the expression of other genes spread throughout the genome. We therefore established the map positions of differentially expressed genes by exploiting previously generated gene mapping datasets. Of the 55 genes highlighted in our comparisons between NILs and recurrent parents, 40 detected eQTL in at least one of the three previous eQTL studies and co-located at the QTL regions, most (83%) having high LOD/LRS scores [22,25] (Table S5). eQTL with high LOD scores have been demonstrated previously to be almost always *cis*-eQTL [22,25,26] placing these genes within the *Rphq*2 or *Rphq*3 QTL regions. The observation that so many significantly differentially expressed genes appeared to be regulated in *cis- *is in agreement with previous studies [25,29]. An exception was unigene 6636, encoding HvERF4. This gene mapped to 71 cM on chromosome 1H, consistent with the position of its rice homologue Os05g41780.1 predicted by conservation of synteny [28]. This observation raises the possibility that the introgressed regions at either *Rphq*2 or *Rphq*3 contain a polymorphic *trans*-acting regulator that differentially modulates expression of *HvERF4*. No eQTL for *HvERF4 *was detected at the regions corresponding to *Rphq*2 or *Rphq*3 in the St/Mx DH mapping population, consistent with the fact that it does not segregate for *Rphq*2 or *Rphq*3.

HvERF4 is a member of a family of plant transcription factors functionally involved in defence signalling pathways related to ethylene, jasmonic acid and abscisic acid. Over-expression of Arabidopsis *AtERF4 *represses the expression of pathogenesis-related (PR) genes such as basic chitinase and beta-1,3-glucanase genes and genes containing a GCC-box [30], the core sequence element of promoters required for responsiveness to ethylene [31]. In our previous experiment with Steptoe and Morex, cultivars with similar but intermediate levels of partial resistance to leaf rust, we also observed that *HvERF4 *was significantly up-regulated by *Ph*-infection but no differential expression (*p *> 0.2) was observed between the parents [25]. Here, *HvERF4 *was induced in *Ph*-infected L94 (susceptible) (FC = 4.42) and Vada (partially resistant) (FC = 2.42) as compared to mock-inoculated controls (Table S5), and the expression level of the Vada allele was only a third (FC = 0.34) of that of the L94 allele after induction. The association of resistance/susceptibility with lower/higher expression of *HvERF4 *appears to be in agreement with the negative regulatory role of HvERF4 on the expression of PR and other defence responsive genes. However, consistent association of higher expression of PR genes with resistance was not observed in Vada and L94. This may reflect the general complexity of natural resistance response coupled with allelic variation at PR genes between these two lines. While this train of inference highlights *HvERF4 *as potentially important in this specific defense interaction, none of the so far reported 20 QTL for partial resistance to leaf rust, nor any of the QTL for resistance to heterologous rusts is co-located with *HvERF4 *at 71 cM on chromosome 1H [11-16]. Thus, *HvERF4 *is not a positional candidate for any of the reported QTL. However, of direct relevance is a previously highlighted eQTL hotspot for 

 genes that was associated with OPA-SNP 11_20157 [25] at 70 cM (98 cM on the consensus map [28]) on chromosome 1H spanning the region containing *HvERF4*. This hotspot comprised 127 eQTL in less than a 10 cM interval and contained genes primarily involved in defence response [25]. Given its known role in PR-protein regulation, we speculate that *HvERF4 *represents a key regulatory relay component of the signalling pathway that controls expression of at least a portion of the genes with eQTL located at the hotspot on chromosome 1H. Considering these observations together we hypothesise that the causal genetic polymorphism at either *Rphq*2 or *Rphq*3 differentially regulates *HvERF4 *in *trans *(possibly through direct or indirect modulation of ethylene, jasmonic acid or abscisic acid levels, known in *Arabidopsis *to alter levels of *AtERF4 *expression [30]), the consequence of which is differential regulation of down-stream defence responses. In this scenario, the candidate genes for *Rphq*2 or *Rphq*3 would be those acting up-stream rather than down-stream of *HvERF4 *and possibly involved directly or indirectly in plant hormone signalling pathways. While we did not find such a candidate from the annotated functions of the QTL-specific and differentially expressed candidates for *Rphq*2 and *Rphq*3, the gene controlling expression of *HvERF4 *may, however, not be differentially expressed between L94 and Vada, may not be on our expression platform (which probably contains less than half of the barley genes) or may not be at the orthologous position in rice. An alternative to identifying the causal gene for *Rphq*2 or *Rphq*3 could be through map-based cloning of the *trans*-eQTL for *HvERF4*.

Marcel *et al*. [18] narrowed down the genetic interval for *Rphq*2 to 0.11 cM corresponding to a physical length of 183 kb in barley (Marcel and Niks unpublished data) and a 69.7 kb syntenic region on rice chromosome 4. Inspection of all predicted genes in the *Rphq*2 syntenic interval in rice identified a cluster of six peroxidase genes and a MAP3K gene [18] as potential candidates because of their functional involvement in defence responses. In this study, we identified four barley genes at *Rphq*2 that were differentially expressed and had homologues located in the syntenic region in rice (Table S5, unigene1852 (no.7), unigene2111 (no.9), unigene13865 (no. 8) and unigene8521 (no. 46)). Unigene1852 and 2111 both encode peroxidases and are within the 0.11 cM interval containing *Rphq*2. The other two, according to the fine mapping data of Marcel *et al*. [18] fell just outside the candidate interval. However, given the frequent breakdown in conservation of synteny, positional candidate gene identification using this approach alone remains problematic. Differential expression in the QTL-NILs identified an additional five candidate genes (Figure 5 and Table S5: no. 2, 3, 19, 21and 51) that were not apparently present in the syntenic region of rice. Two of these encode proteins that are functionally involved in signal transduction (Additional file 5, Table S5, no.19 and 21 encoding a kinase and a receptor-like kinase respectively), one PR protein, and one with homology to human NIPA1, implicated in Prader-Willi/Angelman syndrome 1 [32]. One gene showed no homology to known genes. Thus, these five genes, together with the two peroxidase genes, are potential positional candidates for *Rphq*2. Further refinement of the candidate gene list will require knowledge of the role of these genes in defence response and correlation of transcript levels with resistance/susceptibility. Many more genes (*i.e*. 31) were identified as being differentially expressed and located at the *Rphq*3 region. This is expected given the larger interval of the QTL (28 cM for *Rphq*3 *vs*. 4 cM for *Rphq*2) and *Rphq*3 may, therefore, account for differential expression of most of the genes with unassigned map positions. Functionally, none of the differentially expressed genes at *Rphq*2 or *Rphq*3 appear to be obvious candidates for a regulator of HvERF4.

Many defence genes encoding PR proteins and components of the phenylpropanoid pathway such as phenylalanine ammonia lyase (PAL) were, as would be expected, *Ph*-responsive. PR genes encoding beta-1,3-glucanases, chitinases and thaumatin-like proteins exhibit strong *in vitro *anti-fungal activity [33] and numerous studies have shown that transgenic plants expressing PR-proteins have significant improvement of disease resistance [34-37]. PAL, the first committed enzyme in the phenylpropanoid pathway, is involved in synthesis of both phytoalexins and lignin. Phytoalexins are antimicrobial while lignin synthesis contributes to formation of papillae, which are physical barriers against cell wall penetration by the pathogen [38]. As part of the general response to pathogen infection, few of the genes fell into these categories co-located at the two QTL for partial resistance. One exception, unigene25195, encoding a chitinase (PR3), co-located at *Rphq*2, was *Ph*-responsive and differentially expressed between *Rphq*2 and *Rphq*2. However, it was not prioritized as a candidate for *Rphq*2 since the higher level of gene expression was associated with the susceptibility allele *Rphq*2. Whereas a number of defence genes were activated in response to *Ph*-infection, none was found to be a promising candidate for *Rphq*2 *or Rphq*3. Our results support the notion that components of the general defence response have incremental, rather than deterministic, roles in the outcome of an interaction between a plant and a pathogen [39]. Many attempts to identify genes for disease resistance have highlighted those involved in signal transduction [40,41] or physiological and cellular functions [42,43] rather than defence *per se *[44,45].

*Ph*-infection triggers a broad range of biological responses with defence response genes being significantly over-represented. Of note is a set of genes encoding receptor-like kinase (RLK), receptor-like proteins (RLP), WRKY, MAPK and PR proteins (Additional File 1, Table S1), which form a complete and well-explored defence signalling cascade starting with the perception of PAMPs, activation of WRKY transcription factors and the subsequent induction of PR proteins [3,46]. Our results also suggest that, in the absence of cognate *R *genes to *P. hordei*, plants still mount reactions similar to *R*-gene mediated responses as indicated by the significant up-regulation of genes coding for *R *gene (-like) proteins and marker genes for oxidative burst such glutathione S-transferase and peroxidase. Although no obvious *R *genes were identified as candidates for the QTL in this study, *R *gene-like mediated responses may contribute to basal resistance as a complementary mechanism to PAMP-triggered defence responses. Support for this is provided by observations that resistance QTL are often coincident with the location of *R*-gene homologues [47-51] and that mutated *R *genes can induce a resistance phenotype similar to quantitative resistance controlled by multiple genes [52-54].

One striking characteristic of the responses to *Ph*-infection was the activation of signalling pathways related to a broad range of plant hormones including ethylene, gibberellins, auxin, and brassinosteroid as indicated by the up-regulation of genes encoding ethylene-responsive transcription factors, ACC oxidase, auxin-responsive proteins, brassinosteroid insensitive 1-associated receptor kinase 1 (BAK1), gibberellin receptors and a DELLA protein (Additional File 1, Table S1). All of these hormones have been reported to be involved in plant defence responses [55-57] and various defence pathways are interconnected through hormone-mediated signalling pathways forming complex regulatory networks [55,56,58-60]. Here, the identification of the ethylene-responsive factor *HvERF4 *as a putative link between pathogen perception and response is consistent with a role for differential hormone signalling in partial resistance. Understanding the role of *Rphq*2 of *Rphq*3 in initiating and coordinating the response requires further work.

Substantial overlap of *Ph*-responsive genes was identified in super-susceptible (L94) and partially-resistant (Vada) lines. Over 70% of *Ph*-responsive genes were detected in both L94 and Vada and had the same expression patterns (up- or down-regulation) in both lines. An even higher percentage of overlapping *Ph*-responsive genes (79%) was discovered in both Steptoe and Morex, two cultivars with similar and intermediate level of partial resistance. Given that these lines are genetically diverse, we conclude that barley lines without known cognate *R *genes to *P. hordei *exhibit similar responses at the transcriptional level, and that observed differences are largely quantitative. Similar findings have been observed in the comparison between compatible and incompatible interactions [61-63]. A small proportion (7%) of *Ph*-responsive genes in this study did appear to be resistant/susceptible line-specific and it may be that they determine part of the observed phenotypic differences between lines. However, in *Ph*-infected leaves we found no evidence for their differential expression in the comparisons between the two QTL-NILs and their respective recurrent parents. Therefore, if the variation in resistance, accounted for by *Rphq*2 or *Rphq*3, is regulated at the transcriptional level, these are not strong candidate genes.

We generated a robust expression data set in reciprocal *Rphq*2*/Rphq*3 QTL-NILs at 18 hpi, which is the timepoint previously described as being the most critical during *P. hordei *invasion in barley [25]. However, we realise that transcriptional re-programming in response to pathogen infection is a dynamic and complex process and that defence-associated genes respond to input stimuli with different timing and amplitude. A limitation of our experiment is, therefore, that defence response scenarios constructed on the transcriptional profiles of the 802 *Ph*-responsive genes identified here is simply a snapshot of a dynamic process, at the point when infection hyphae have just attempted penetration of the host cells forming haustoria [25]. To extend our understanding of the complex regulatory mechanisms occurring during defence against *P. hordei*, a more comprehensive investigation would involve sampling at multiple timepoints covering the whole infection period.

## Conclusions

Differential expression with QTL-NILs identifies genes predominantly located at the target region(s) providing both transcriptional and positional candidate genes underlying the QTL. Positional analysis of the differentially expressed genes relative to the QTL has the potential to discover regulatory relays initiated from genes within the QTL.

## Methods

### Plant materials

The plant materials used in this study included both recurrent parental lines L94 (highly susceptible to *P. hordei*) and Vada (high level of partial resistance to *P. hordei*) and the QTL-NIL named L94-*Rphq*2*+3 *and Vada-*Rphq*2*+*3 according to the introgressed resistance/susceptibility QTL alleles. Gene symbol '*Rphq' *refers to the resistance allele of the QTL, *i.e*. the allele contributed by Vada, and '*rphq' *refers to the susceptibility L94 allele. Neither of these cultivars carries a cognate *R*-gene to *P. hordei*. The NIL 'L94-*Rphq*2+3' was previously developed through a marker-assisted backcross programme by incorporating leaf rust resistance alleles *Rphq*2 and *Rphq*3 from Vada into L94 susceptible genetic background, whereas the NIL Vada-*rphq*2+3 was generated by reciprocally incorporating the corresponding susceptibility QTL alleles *rphq*2 and *rphq*3 from L94 into Vada genetic background [18]. The resulting resistance levels (relative latency period in hours) of the NILs are 120 ± 1.77 for L94-*Rphq*2*+*3, 106 ± 2.54 for Vada-*Rphq*2*+*3, as compared to 100 ± 1.77 for L94 and 127 ± 1.80 for Vada [18]. The genetic lengths of the two introgression segments on chromosome 2H were 4.6 cM for *Rphq*2 and 4.4 cM for *Rphq*2; the two QTL segments on chromosome 6H were 22.6 cM for *Rphq*3 and 45.8 cM for *rphq*3 [18].

Plant growth and leaf inoculations were performed as previously described [25]. The parental lines L94 and Vada and their QTL-NILs, each with 10 seedlings were grown in one tray (37 × 39 cm) in two rows 30 cm apart. A total of eight trays were prepared, with four each used as biological replicates for pathogen inoculation and mock inoculation. The plant growth conditions were as described by Chen *et al*. [25].

### Pathogen inoculation

Inoculation with *P. hordei *isolate 1.2.1 was performed on 9-day old seedlings when the first leaf was fully developed and the second leaf was emerging. Leaves were laid horizontal and gently fixed over the soil prior to inoculation. The inoculation was described in Chen *et al*. [25]. Per plant tray, 8 mg of urediospores plus 32 mg of *Lycopodium *spores (added as a carrier) were thoroughly mixed by vortexing and applied to the adaxial sides of the seedling leaves using a settling tower inoculation facility. This amount of spores corresponds to a deposition of about 500 spores per cm^2^. Mock inoculation of parental lines was carried out using 40 mg of *Lycopodium *spores only. All trays were transferred to a dark dew chamber at 18°C and 100% relative humidity for 10 hours overnight, before being placed in the glasshouse for infection development.

### Leaf sampling

At 18 hpi, both pathogen- and mock-inoculated leaf blades of each replicate and treatment were collected separately into falcon tubes and immediately flash-frozen in liquid nitrogen before being stored at -80°C until use.

### RNA isolation, labelling and microarray platform

RNA isolation was done using the TRIZOL^® ^reagent according to the manufacturer's protocol. cDNA synthesis, labeling and hybridization were performed following the optimized protocol developed by the Sequencing & Microarray Facility at SCRI. The Agilent 8 × 15 k format custom array system was used as the platform for RNA profiling. Detailed protocols are described in Chen *et al*. [25].

### Sample layout on the 8 × 15 k Agilent arrays

The Agilent platform may be used as a two-colour microarray system allowing two differentially-labeled samples to be tested on a single array. We used three different sample layouts depending upon the biological questions to be addressed: 1) RNA samples from *Ph*-infected parents and mock-inoculated controls (four replicates) were hybridized onto single arrays to identify *Ph*-responsive genes (array slide 1 in Table 2); 2) RNA samples from *Ph*-infected L94 and Vada (four replicates) were hybridized onto single arrays to test genome-wide differential expression (slide 2 in Table 2); 3) RNA samples from *Ph*-infected L94 and L94-*Rphq*2*+*3 or Vada and Vada-*Rphq*2*+*3 were put on single arrays with four replicates (8 arrays) (slide 3 in Table 2) to compare expression levels of parental lines with their respective NILs. In all sample layouts, a balanced dye swap strategy was applied as indicated in the Table 2.

**Table 2:** Microarray experimental design:

Array slide	Replicate	Sample pairs					
		
		Name	Treatment	Label	Name	Treatment	Label
1	I	L94	Mock	C3	L94	*Ph*-infected	C5
1	I	Vada	Mock	C3	Vada	*Ph*-infected	C5
1	II	L94	Mock	C3	L94	*Ph*-infected	C5
1	II	Vada	Mock	C3	Vada	*Ph*-infected	C5
1	III	L94	Mock	C5	L94	*Ph*-infected	C3
1	III	Vada	Mock	C5	Vada	*Ph*-infected	C3
1	IV	L94	Mock	C5	L94	*Ph*-infected	C3
1	IV	Vada	Mock	C5	Vada	*Ph*-infected	C3

2	I	L94	*Ph*-infected	C3	Vada	*Ph*-infected	C5
2	II	L94	*Ph*-infected	C5	Vada	*Ph*-infected	C3
2	III	L94	*Ph*-infected	C3	Vada	*Ph*-infected	C5
2	IV	L94	*Ph*-infected	C5	Vada	*Ph*-infected	C3

3	I	L94	*Ph*-infected	C3	L94-*Rphq*2*+*3	*Ph*-infected	C5
3	I	Vada	*Ph*-infected	C3	Vada- *Rphq*2*+*3	*Ph*-infected	C5
3	II	L94	*Ph*-infected	C5	L94- *Rphq*2*+*3	*Ph*-infected	C3
3	II	Vada	*Ph*-infected	C5	Vada- *Rphq*2*+*3	*Ph*-infected	C3
3	III	L94	*Ph*-infected	C3	L94- *Rphq*2*+*3	*Ph*-infected	C5
3	III	Vada	*Ph*-infected	C3	Vada- *Rphq*2*+*3	*Ph*-infected	C5
3	IV	L94	*Ph*-infected	C5	L94- *Rphq*2*+*3	*Ph*-infected	C3
3	IV	Vada	*Ph*-infected	C5	Vada- *Rphq*2*+*3	*Ph*-infected	C3

Array slide 1: *Ph*-infected *vs*. mock-inoculated controls for *Ph*-responsive genes; Array slide 2: *Ph*-infected L94 *vs. Ph*-infected Vada for differentially expressed genes; Array slide 3: *Ph*-infected parents *vs. Ph*-infected QTL-NILs for QTL specific and differentially expressed genes.

### Deposition of microarray data

The raw microarray data and relevant experimental metadata, which are MIAME (Minimum Information About a Microarray Experiment) compliant, are deposited at the ArrayExpress microarray data archive http://www.ebi.ac.uk/microarray-as/ae/ at the European Bioinformatics Institute (accession numbers: E-TABM-980).

### Data extraction, normalisation and significance criteria for differential expression

Data extraction and normalisation were done independently for the three different experiments with GeneSpring (v.7.3) software as described previously [25]. Briefly, dye swap was corrected in relevant samples, followed by Lowess (LOcally WEighted polynomial regreSSion) normalisation to minimize differences in dye incorporation efficiency in a two-channel microarray platform [64]. Differentially expressed genes were first selected on fold change >2 followed by a Students *t*-test on log-transformed normalised ratio data, setting the False Discovery Rate (FDR) to 0.05.

## Abbreviations

*Ph*: *Puccinia hordei*; QTL: quantitative trait loci; eQTL: expression QTL; QTL-NIL: QTL-specific nearly isogenic line; RIL: recombinant inbred line, PAMP: pathogen-associated molecular pattern; PTI: PAMP-triggered immunity; ETI: effector-triggered immunity, FDR: false discovery rate; GO: gene ontology; LOD: log of odds; LRS: likelihood ratio statistics; FC: fold change; SFP: single feature polymorphism; TDM: transcript derived marker.

## Authors' contributions

Conceived and designed the experiments: XC, REN, AD and RW; performed the experiments: XC; wrote the paper: XC and RW. Pathogen infection and sampling: REN, XC, TCM and AV. Microarray and data deposition: PH and JM. All authors read and approved the final manuscript.

## Supplementary Material

Additional file 1 **Table S1**. Expression information of *Ph*-reponsive genes identified on L94 and Vada (*Ph*-infected *vs*. mock control). Click here for file

Additional file 2 **Table S2**. Expression information of resistant/susceptible line-specific and *Ph*-responsive genes. Click here for file

Additional file 3 **Table S3**. Expression of the resistant/susceptible line-specific genes (upper/lower panel) reproduced as *Ph*-responsive genes in St and Mx. Click here for file

Additional file 4 **Table S4**. Genome-wide differentially expressed genes in *Ph*-infected seedlings between Vada and L94. Click here for file

Additional file 5 **Table S5**. List of the 55 differentially expressed genes showing expression ratios and *p*-values in different comparisons and map position of eQTL and corresponding genes from different sources. Click here for file
